# Monkfish (*Lophius litulon*) Peptides Ameliorate High-Fat-Diet-Induced Nephrotoxicity by Reducing Oxidative Stress and Inflammation via Regulation of Intestinal Flora

**DOI:** 10.3390/molecules28010245

**Published:** 2022-12-28

**Authors:** Xiangyu Ren, Bingtao Miao, Hongjie Cao, Xiaoxiao Tian, Lujia Shen, Zuisu Yang, Falei Yuan, Yaping Ding

**Affiliations:** 1 Zhejiang Provincial Engineering Technology Research Center of Marine Biomedical Products, School of Food and Pharmacy, Zhejiang Ocean University, Zhoushan 316022, China; 2 Department of Nutrition, Zhoushan Hospital of Traditional Chinese Medicine (Affiliated to Zhejiang University of Traditional Chinese Medicine), Zhoushan 316000, China

**Keywords:** monkfish peptides, kidney, high-fat diet, nuclear factor erythroid 2-related factor 2, nuclear factor-kappa B, intestinal flora

## Abstract

Background: Renal damage and intestinal flora imbalance due to lipotoxicity are particularly significant in terms of oxidative stress and inflammation, which can be alleviated with bioactive peptides. The monkfish (*Lophius litulon*) is rich in proteins, which can be used as a source of quality bioactive peptides. This study aimed to examine the protective effect of monkfish peptides on renal injury and their potential role in regulating gut microbiota. Methods: Monkfish meat was hydrolyzed using neutral protease and filtered, and the component with the highest elimination rate of 2,2-diphenyl-1-picrylhydrazyl was named *lophius litulon* peptides (LPs). Lipid nephrotoxicity was induced via high-fat diet (HFD) feeding for 8 weeks and then treated with LPs. Oxidative stress, inflammatory factors, and intestinal flora were evaluated. Results: LP (200 mg/kg) therapy reduced serum creatinine, uric acid, and blood urea nitrogen levels by 49.5%, 31.6%, and 31.6%, respectively. Renal vesicles and tubules were considerably improved with this treatment. Moreover, the activities of superoxide dismutase, glutathione peroxidase, and total antioxidant capacity increased significantly by 198.7%, 167.9%, 61.5%, and 89.4%, respectively. LPs attenuated the upregulation of HFD-induced Toll-like receptor 4 and phospho-nuclear factor-kappa B and increased the protein levels of heme oxygenase 1, nicotinamide quinone oxidoreductase 1, and nuclear factor erythroid 2-related factor 2. The dysbiosis of intestinal microbiota improved after LP treatment. Conclusions: LPs significantly improve antioxidant activity, reduce inflammatory cytokine levels, and regulate intestinal dysbiosis. Thus, LPs are potential compounds that can alleviate HFD-induced renal lipotoxicity.

## 1. Introduction

As living standards improve, the long-term consumption of a high-fat diet (HFD) and inadequate exercise increases health and obesity-related metabolic disease risks [1]. Long-term HFD may lead to chronic kidney disease (CKD) in addition to nonalcoholic fatty liver disease, dyslipidemia, metabolic syndrome, type 2 diabetes, hypertension, and insulin resistance [2,3,4]. As Moorhead et al. explained in 1982 [5], the lipid nephrotoxicity hypothesis stimulated many studies on lipid metabolism in renal diseases. These studies suggested that lipid accumulation causes glomerular membrane hyperplasia and epithelial cell damage, leading to the development of renal diseases [6]. Recent research has demonstrated that through lipotoxicity and imbalance in the relationship between reactive oxygen species (ROS) and inflammation, dyslipidemia, a crucial component of the multihit mechanism, can harm healthy kidneys [7]. Thus, it is speculated that lipid-accumulation-induced precursor disorders may elicit oxidative stress or inflammation, which is necessary to induce lipid-mediated kidney damage; however, molecular mechanisms involved in this process remain unclear.

Oxidative stress and inflammation are correlated, and both contribute to the development of renal diseases [8]. Hyperlipidemia can greatly augment ROS production in monocytes [9], and inflammatory mediators are ROS activators in the kidney. These ROS activators stimulate macrophages to produce free oxygen radicals, and improper ROS production can induce stress and lead to tissue dysfunction. To resist oxidative stress, the nuclear factor erythroid 2-related factor 2/Kelch-like ECH-associated protein 1 (Nrf2/Keap1) signaling pathway activates the genes for producing antioxidant enzymes [10]. In general, enzymes, such as catalase and the superoxide gasification enzyme, reduce ROS accumulation. Nrf2, a transcription factor that controls the expression of certain downstream proteins, such as nicotinamide quinone oxidoreductase 1 (NQO1) and heme oxygenase-1 (HO-1), is released to combat ROS [11]. Interleukin (IL)-1 and tumor necrosis factor (TNF) stimulate Toll-like receptors (TLRs) to activate IκB kinase (IKK), thereby activating nuclear factor-kappa B (NF-κB) (p65) [12,13]. The modulation of the Nrf2/Keap1 and TLR4/NF-κB signaling pathways is a crucial strategy in the control of HFD-induced oxidative stress and inflammatory kidney injury. 

Intestinal flora participates in critical processes that control metabolism, inflammation, and immunity, making it critical for preserving human health and preventing disease [14]. Dietary differences can lead to changes in the intestinal flora, particularly in obese patients [15]. The consumption of different foods affects the growth and reproduction of certain intestinal flora in the body. For instance, long-term HFD decreases the diversity of microorganisms but increases the proportion of *Firmicutes* and *Bacteroides* [16]. A growing body of evidence indicates that the intestinal and renal systems interact. Intestinal flora can improve kidney function [17], and kidney metabolism can alter the variety and abundance of gut microbiota. Patients with uremia have 100-fold more intestinal aerobes (*Enterobacter*, *Enterococcus*, and other species) and significantly lower anaerobic bacteria than healthy people [18]. It has been shown that both the external living environment and the host’s lifestyle (diet, stress, disease, and others) affect the diversity and function of intestinal flora, thereby affecting human health [19]. Diet is regarded as a critical lifestyle factor that affects intestinal flora, and dietary practices and caloric intake, in particular, may have a considerable impact on the inter-individual diversity of gut bacterial strains [20,21,22]. Therefore, the concept of the kidney–gut axis was proposed according to the interaction between the two [23], and the intestinal microbiota is a key factor in the kidney–gut axis [24]. 

Owing to their anti-tumor, anti-hypertensive, anti-oxidant, and anti-bacterial capabilities, marine active peptides have gained increasing attention in recent years [25]. The advantageous anti-inflammatory properties of marine natural products are linked to their distinctive chemical composition. They can produce anti-inflammatory effects via the immune system, intestinal microecology, and autophagy system and through the conventional TLR4/NF-κB signaling route [26]. Studies have shown that deep-sea fish collagen peptides can improve glucose and lipid metabolic dysfunction in patients with type 2 diabetes by increasing insulin sensitivity and reducing renal marker proteins in the urine, such as urinary proteins, activating mitochondrial autophagy in the skin to rescue mitochondrial dysfunction [27,28,29]. Monkfish, a type of deep-sea fish, inhabits the Atlantic, Pacific, and Indian Oceans. Tian et al. [30] discovered that bioactive peptides from monkfish (*Lophius litulon*) produced high antioxidant activity and decreased H_2_O_2_-induced oxidative damage in RAW264.7 cells. Moreover, Ye et al. [31] found that monkfish peptides can increase liver antioxidant capacity and alleviate HFD-induced non-alcoholic fatty liver disease. Therefore, it was hypothesized that monkfish peptides inhibit the NF-κB pathway, activate the Nrf2 pathway, and lower oxidative stress and inflammatory response in addition to controlling the composition of intestinal flora to alleviate chronic kidney damage. Due to the properties of monkfish, active ingredients are removed from the meat to increase the value of monkfish and utilize protein resources more effectively. Using mice with existing lipid nephrotoxicity, the effect of LPs on renal health was also evaluated.

## 2. Results

### 2.1. Identification and Protein Analysis of LPs (<1 kDa)

The component with the greatest 2,2-diphenyl-1-picrylhydrazyl clearance rate was identified using the ultrafiltration separation of monkfish hydrolysate dissolved in neutral protease. As shown in Table 1, there were relatively high levels of hydrophobic amino acid (HAA) and negatively charged amino acids (NCAAS) in LPs, which measured 25.74 (g/100 g) and 15.59 (g/100 g), respectively. The essential amino acids present in LPs were 28.43 g per 100 g without detectable levels of tryptophan, asparagine, or glutamine in acidic conditions. De novo sequencing identified 3276 free peptides from 98 LPs obtained via database matching on the basis of our research group’s predetermined polypeptide sequence [31]. No overlap was found between the results and free peptide data obtained via database comparison. According to the findings, LPs have a significant antioxidant capacity and nutritional value.

### 2.2. Effect of LPs on the Renal Index of Mice 

To evaluate the success of HFD modeling, changes in mouse renal weight were evaluated. Renal weight change is a remarkable feature of lipid accumulation. In Table 2, the kidney index of the mice with lipotoxicity increased by 6% (*p* < 0.05), whereas dietary additions of LPs to the diet reduced the renal index by 4% and 6%, respectively. This suggests that LPs can reduce kidney damage induced by HFD. 

### 2.3. Effect of LPs on the Renal Function of Mice 

To investigate the protective effect of LPs on renal injury induced by HFD, the levels of uric acid (UA), creatinine (CRE), and blood urea nitrogen (BUN) were measured. It is important to monitor the levels of blood BUN, CRE, and UA as in-depth clinical indicators of renal function. As shown in Table 3, it is believed that HFD can contribute to renal deterioration, as the serum levels of UA, CRE, and BUN in the HFD group were 118%, 65%, and 96% higher, respectively, than in the ND group. In mice after high-dose LP therapy, UA, CRE, and BUN levels decreased by 49%, 32%, and 32%, respectively, suggesting that LPs could reverse renal impairment caused by HFD.

### 2.4. Effect of LPs on Renal Histomorphology of Mice

**Figure 1:**
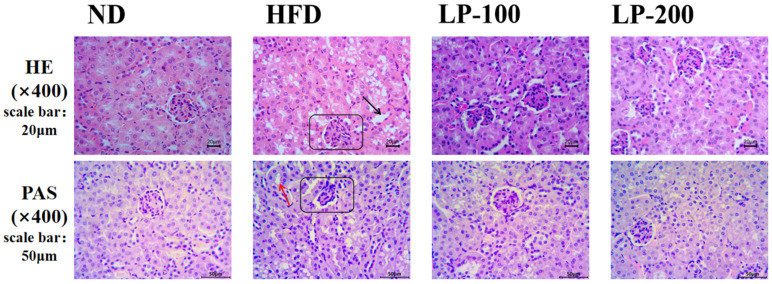
Effect of LPs on the morphological characteristics of kidney in HFD-fed mice. PAS trichrome and H&E staining were applied to kidney sections.

Figure 1 shows an optical microscope observation of hematoxylin and eosin (H&E) staining and periodic acid-Schiff (PAS) staining on renal pathological processes induced by LPs. H&E staining showed that renal corpuscles in the ND group comprised renal vesicles wrapped around the glomerulus, with clear renal vesicles and regular renal tubules and without inflammatory infiltration. However, in the HFD group, glomerular atrophy, cystic enlargement, unclear structure of proximal convoluted tubules in renal tubules, and obvious vacuolar degeneration damage were observed, and inflammatory cell infiltration around the tubules was detected. The structure of renal corpuscles and tubules was improved after LP treatment, especially in the LP-200 group.

The PAS staining of the kidney stained the purplish red part of renal interstitium. The renal histopathology of mice in the ND group was normal, and mesangial hyperplasia was not observed. Moreover, in the ND group, the mesangial area was small and the glomerular morphology was normal. In the HFD group, the positive staining of kidney was significantly increased, and the basal membrane around renal tubules was significantly thickened. However, with the increase of LP dose, the positive sites in the glomerulus of mice in the treatment group were significantly reduced, and the thickness of the basal membrane around the renal tubules was reduced.

### 2.5. Effect of LPs on Antioxidant Levels in Mouse Kidneys

To investigate whether LPs could affect oxidative stress levels in mice, the levels of antioxidant enzymes and malondialdehyde (MDA) in the kidney tissues of mice were evaluated. As shown in Table 4, in the HFD group, the content of MDA was increased by 108%, while a reduction of 55% in catalase (CAT), 71% in superoxide dismutase (SOD), 48% in total antioxidant capacity (T-AOC), and 66% in glutathione peroxidase (GSH-Px) was observed, indicating that the antioxidant system in the mice was damaged, which was in the state of oxidative stress. After high-dose LP treatment, the levels of MDA decreased by 43%, whereas those of T-AOC, SOD, GSH-Px, and CAT increased by 62%, 198%, 168%, and 89%, respectively. It can be seen that LPs have a remarkable effect on reducing oxidative stress caused by HFD in mice kidneys.

### 2.6. Effect of LPs on Inflammatory Factors in the Mice’s Kidneys

When renal function is impaired, the glomerular mesangial cells produce a large amount of interleukin-6 (IL-6), interleukin-1β (IL-1β), and other inflammatory mediators, exacerbating glomerulosclerosis and renal function decline [32]. To test the effect of LPs on the secretion of inflammatory factors in the kidney, IL-6, IL-1β, and tumor necrosis factor-α (TNF-α) levels in the mouse kidney were detected. In Table 5, the levels of IL-1β, IL-6, and TNF-α in the kidney of the HFD group were increased by 55%, 31%, and 196%, respectively. After high-dose LP treatment, the production of all three factors decreased by 27%, 21%, and 41%, respectively, suggesting that LPs inhibit the release of inflammatory factors and alleviate renal injury induced by HFD.

### 2.7. Effect of LPs on Expression of Nrf2-Pathway-Related Proteins

**Figure 2:**
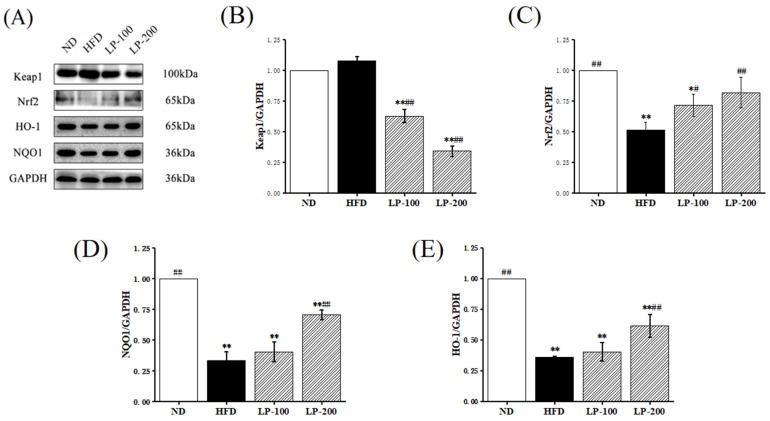
Effect of LPs on the antioxidant response mediated by Nrf2 in HFD-fed mice (**A**–**E**). Western blot showed the protein expression levels of Keap1, Nrf2, HO-1, and NQO1 in the renal lysates of mice fed with CD, CD+HFD, and CD+HFD+LPs. The signal intensity was quantitatively analyzed. * *p* < 0.05, ** *p* < 0.01 vs. ND group; # *p* < 0.05, ## *p* < 0.01 vs. HFD group.

The Nrf2 signal transduction pathway is a classic oxidative stress signal transduction pathway, primarily involving Nrf2, Keap1, HO-1, and NQO1. To explore the specific alleviating mechanism of LPs in kidney injury, the Nrf2/Keap1 signaling pathway was further studied. In Figure 2, in the HFD group, Nrf2, HO-1, and NQO1 levels were reduced by 48%, 64%, and 67%, respectively. However, high-dose LP treatment increased the levels of these proteins by 58%, 112%, and 71%, respectively. The Keap1 protein level was increased by 8% in the HFD group, and significantly decreased after LP treatment. These results suggested that the protective mechanism of LPs is associated with the reactivation of the Nrf2/Keap1 signaling pathway.

### 2.8. Effect of LPs on Expression of NF-κB-Pathway-Related Pathway Proteins

**Figure 3:**
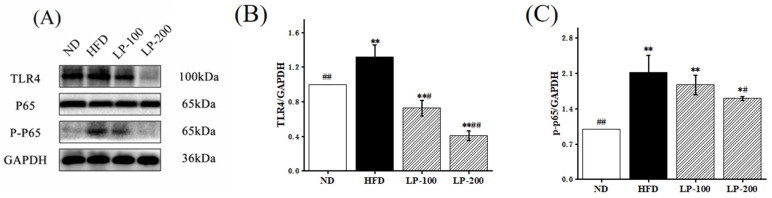
Effect of LPs on TLR4/NF-κB inflammatory pathway in HFD-fed mice. (**A**–**C**) Western blot. The expression levels of TLR4, p65, and p-p65 proteins in the renal lysates of mice fed with CD, CD+HFD, and CD+HFD+LPs. The signal intensity was quantitatively analyzed. * *p* < 0.05, ** *p* < 0.01 vs. ND group; # *p* < 0.05, ## *p* < 0.01 vs. HFD group.

TLR4/NF-κB is a crucial signaling pathway in inflammatory stress, primarily involving TLR4, NF-κB (p65), and phospho-NF-κB (p-p65). To further understand the protective mechanism of LPs on lipotoxicity, we examined the NF-κB inflammatory protein pathways. In Figure 3, the protein expressions of TLR4 and p-p65 in the kidney of the HFD group were increased by 32% and 112% in comparison with the ND group. LP treatment improved the expression level, suggesting that LPs inhibit the TLR4/NF-κB signaling pathway. 

### 2.9. Sequencing Results of Intestinal Microflora 

**Figure 4:**
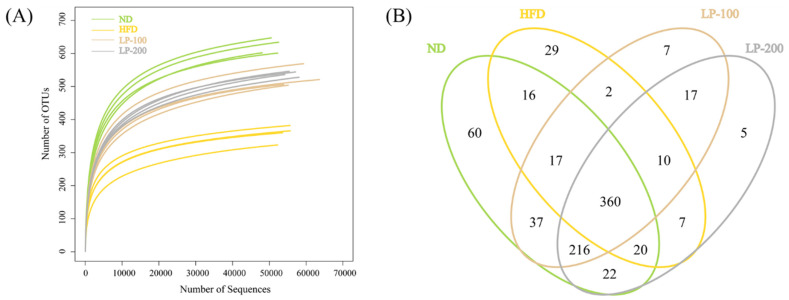

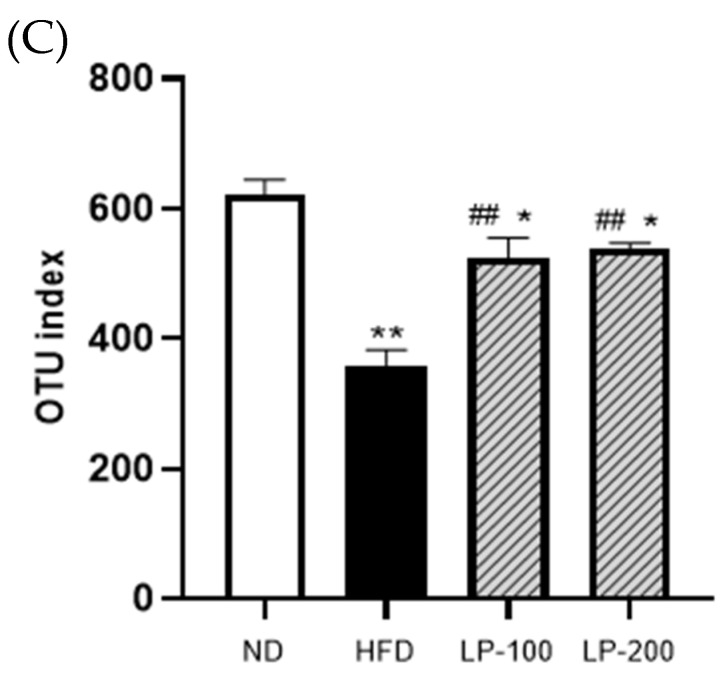
Dilution curves (**A**), Venn diagram (**B**), and OTU index (**C**) of intestinal flora samples in each group. * *p* < 0.05, ** *p* < 0.01 vs. ND group; ## *p* < 0.01 vs. HFD group.

The dilution curve of this experiment is shown in Figure 4A. Until there are 5000 sequences, the curve begins to flatten out, showing that all species in the sample have been covered by the sequencing depth. At this point, Figure 4C shows that the operational taxonomic unit (OTU) values of the ND and LPs groups were significantly higher than those of the HFD group. As shown in Figure 4B, it was determined that there are 825 OTUs in total. By comparison, 360 OTUs were found in the four groups, and 216 OTUs were found in the LP and ND groups. By comparison, LP-200 and HFD groups had at least seven OTUs.

### 2.10. α-Diversity Analysis of Intestinal Flora

**Figure 5:**
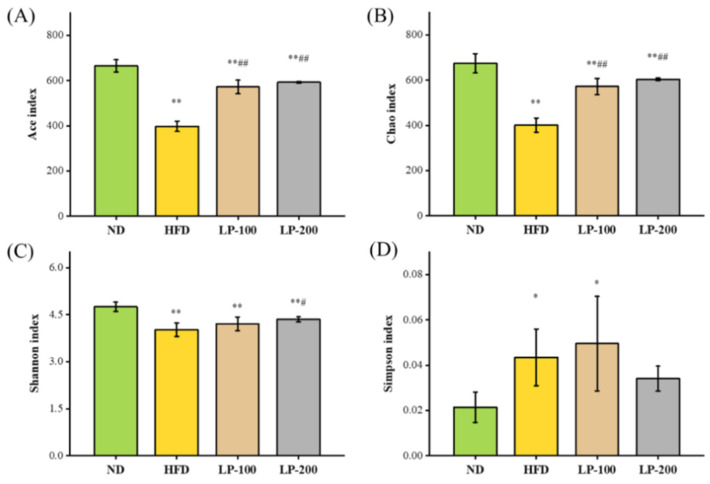
Comparison of the α-diversity index of the mice’s gut flora for each group (**A**) Chao index, (**B**) Ace index, (**C**) Shannon index, and (**D**) Simpson index. * *p* < 0.05, ** *p* < 0.01 compared with ND; # *p* < 0.05, ## *p* < 0.01 compared with HFD.

The higher Shannon, Ace, and Chao indices and lower Simpson indexes indicate greater species diversity. The study results revealed that although its Simpson index was significantly higher (*p* < 0.05), the Chao, Ace, and Shannon indices of the HFD group were reduced significantly compared with those of the ND group (*p* < 0.01), (Figure 5A–D). Shannon and Simpson in the LP-200 group were not significantly different, while the Chao and Ace in the LPs group were significantly increased (*p* < 0.01) in comparison with the HFD group. Shannon index in the LP-200 group was significantly increased (*p* < 0.05), while Simpson index was not. These results suggested that LPs restored the diversity of gut flora in mice, which was decreased by HFD.

### 2.11. β-Diversity Analysis and UPGMA Analysis of Intestinal Microbiota in Mice

**Figure 6:**
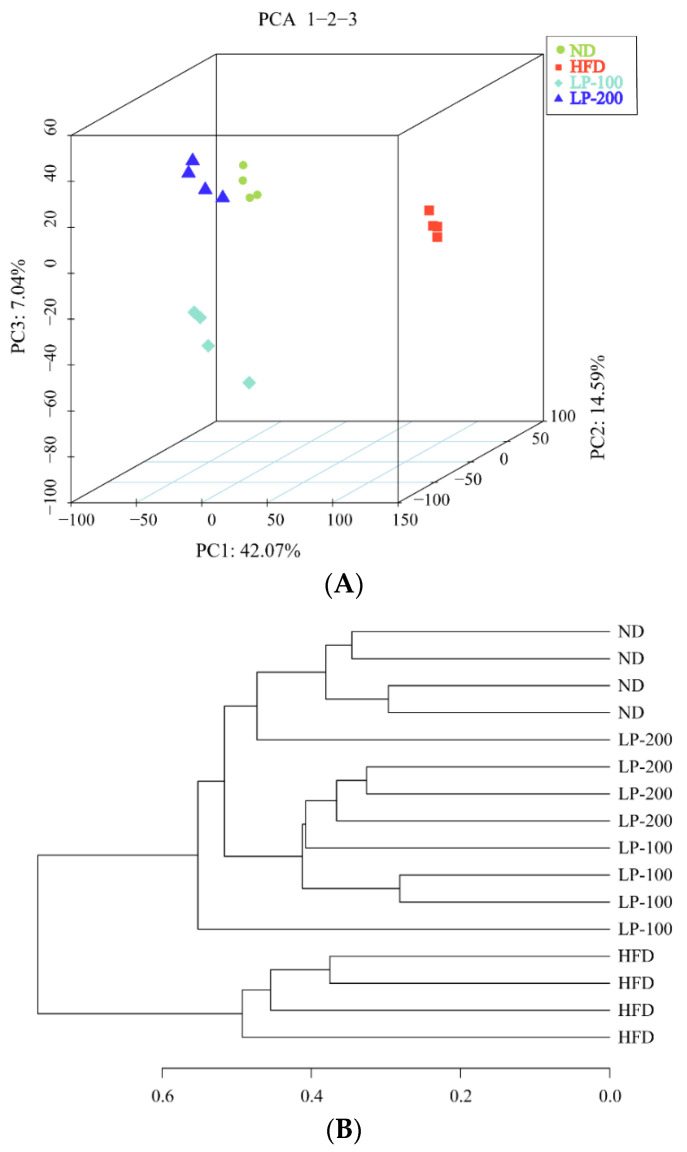
PCOA (3D) illustration of the non-weighted UniFrac-based principal coordinate analysis of the flora in the digestive tract (**A**). Diagram showing the gut flora based on Jaccard-based UPGMA analysis (**B**).

To compare variations in species diversity, β-diversity analysis was performed. In this study, the unweighted UniFrac index was measured. In the analysis graph obtained using this index, the farther the distance between samples, the greater the diversity difference between samples. In this paper, the β-diversity of each group was analyzed. As shown in the PCOA diagram of principal coordinate analysis in Figure 6A, the LPs group and ND group were close to each other, while the HFD group and ND group and LPs group were far apart, especially the LP-200 group and ND group. These findings imply that HFD modified the intestinal microflora structure of mice and that the diversity difference between the LP and ND groups was small, whereas the diversity difference among the HFD, ND, and LP groups was large. As shown in Figure 6B, the evolutionary distance between each group of intestinal flora samples can be differentiated by UPGMA graph tree branch distance and cluster distance. The LP and the ND groups were in the same branch, and the LP-200 group was close to the ND group. The HFD, LP, and ND groups were distributed in different branches of the cluster tree. These findings suggest that LPs can help with the gut microbiota structure of HFD-fed mice. 

### 2.12. Mice Intestinal Microbiota Analysis Using Metastats

**Figure 7:**
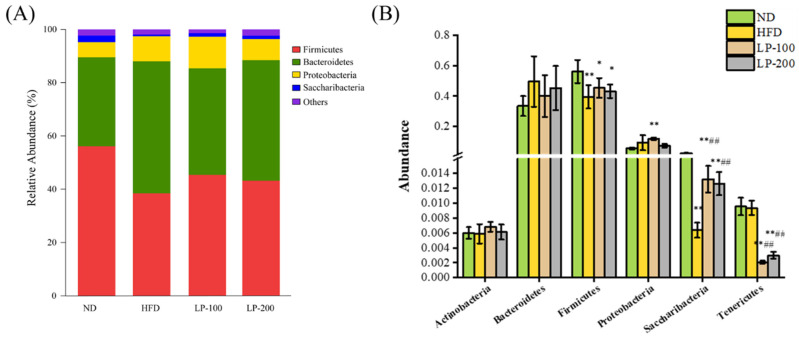
Comparison of histogram (**A**) and taxonomic differences in the bacterial species distribution (**B**) at the phylum level of mouse intestinal bacteria. * *p* < 0.05, ** *p* < 0.01 compared with ND; ## *p* < 0.01 compared with HFD.

Differentiations in bacterial taxonomic composition among groups were examined using Metastats to comprehend variations in microbial community abundance. According to the findings (Figure 7), at the phylum level, *Firmicutes* and *Bacteroidetes* dominated the gut flora of mice in each group. Compared with the ND group, *Bacteroidetes* and *Proteobacteria* were increased, and *Firmicutes* and *Saccharibacteria* were decreased in the intestinal flora of the HFD group (*p* < 0.05). *Actinobacteria* and *Tenericutes* also decreased in this group, reflecting the imbalance of intestinal microecology in mice. Compared with the HFD group, *Saccharibacteria* was increased in the gut flora of LP-treated mice (*p* < 0.01). Moreover, *Bacteroidetes* was decreased, and *Firmicutes* was increased in this group. These findings indicate that in mice fed an HFD, LPs could control the phylum level composition of gut flora.

### 2.13. Lda Effect Size (LEfSe) Analysis of Intestinal Microflora in Mice

**Figure 8:**
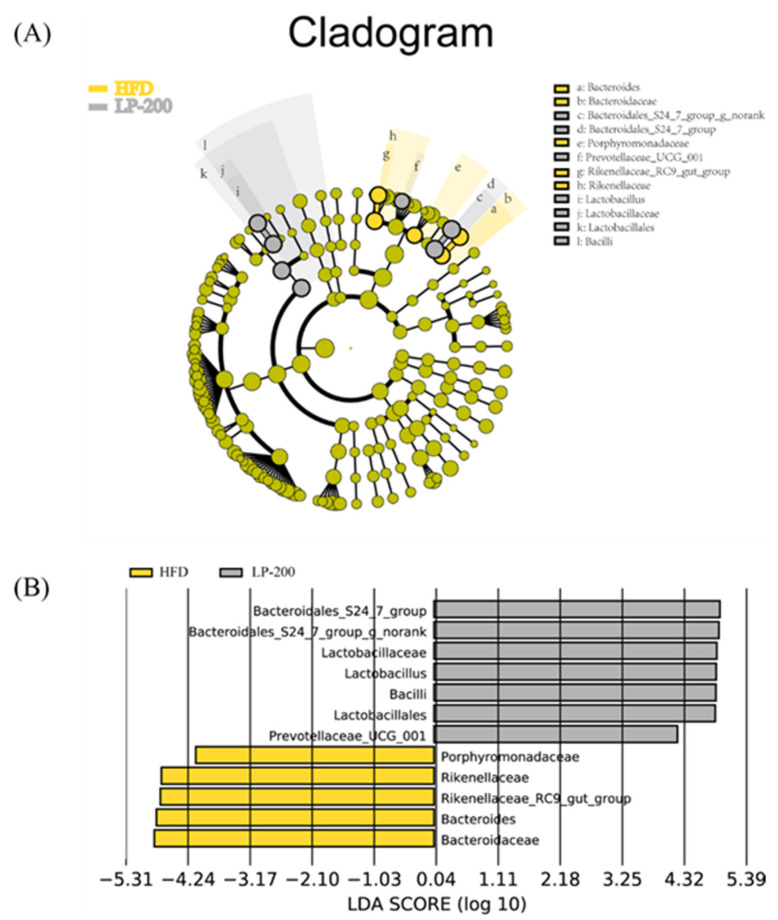
Cladogram (**A**) and LEfSe analysis (**B**) of mouse intestinal flora.

To evaluate variations in intestinal microbiota in several mice groups, an LEfSe analysis on the basis of OTU levels was performed. As shown in Figure 8, at the species level, LPs treatment effectively reduced *Bacteroidaceae, Bacteroides, Rikenellacea–RC9–gut group*, and *Bacteroidaceae* and the relative abundance of *Rikenellaceae* and *Porphyromonadaceae*. The addition of the *Bacteroidales–S24–7 group*, *Lactobacillaceae*, *Lactobacillus, Bacilli,* and *Lactobacillales* are relatively abundant. These findings indicate that gut flora in HFD-fed mice were influenced by LPs with respect to their shape and composition. 

### 2.14. Functional Prediction of Intestinal Microbiota in Mice

**Figure 9:**
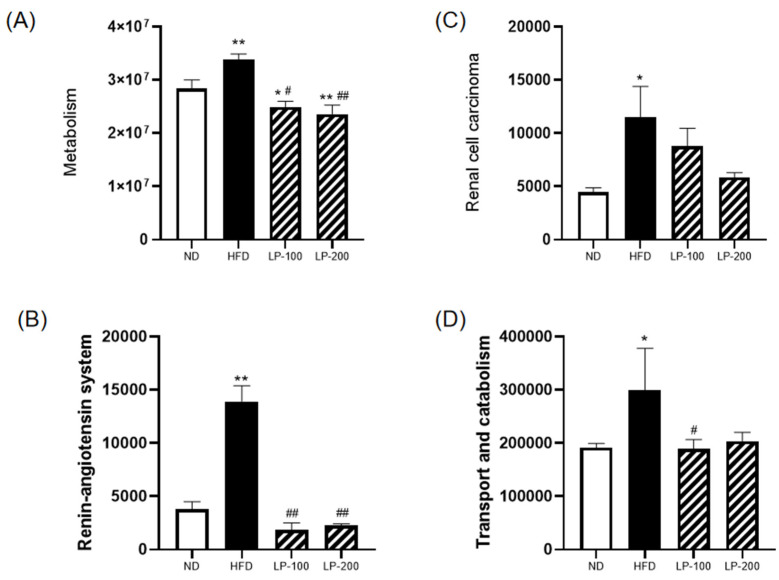
Functional prediction of intestinal microbiota in mice. (**A**) Metabolism, (**B**) renin–angiotensin system, (**C**) renal cell carcinoma, and (**D**) transport and catabolism. * *p* < 0.05, ** *p* < 0.01 compared with ND; # *p* < 0.05, ## *p* < 0.01 compared with HFD.

A functional study and gut microbiota prediction were performed to better understand the correlation between the mouse gut microbiota and the kidneys. According to Figure 9A–D, changes in intestinal flora brought on by a long-term HFD can have varying degrees of effects on metabolism, renal cell carcinoma, the renin–angiotensin system, and the transport and catabolism systems. However, after LP administration, the body’s performance was comparable to that of the control group.

## 3. Discussion 

The present study examined the effects of LPs on the alleviation of renal damage using an HFD-induced renal damage model. HFD up-regulated the levels of BUN, CRE, UA, and inflammatory-cell-related factors, whereas it down-regulated the activity of antioxidant enzymes. These results suggest that HFD alters the body’s metabolic balance and regulates glucose and lipid metabolism, ultimately causing hyperlipidemia, lipid accumulation in the kidney, increased systemic inflammatory cytokine production, and kidney damage. LP therapy significantly improved the kidney function of HFD-fed mice. Meanwhile, H&E and PAS staining showed improvement in renal pathology. Both biochemical and histopathological assessments revealed that LPs ameliorated HFD-induced chronic kidney injury in mice. Thus, the present study confirmed the feasibility of LPs for treating metabolic diseases caused by obesity and could provide a basis for the study of such diseases. Previous research demonstrated that the excessive intake of a fat-rich diet may lead to hyperlipidemia, dyslipidemia, systemic oxidative stress, and inflammation, particularly leading to CKD and nonalcoholic fatty liver disease [33,34,35,36]. Meanwhile, the long-term intake of HFD leads to liver and kidney dysfunction in addition to lipid accumulation in the kidney [37]. 

Inflammation is a major factor in most CKDs, and inflammatory factors play a crucial role in inflammation. Inflammatory stress promotes renal lipid accumulation and glomerular lesions, and renal injury is positively correlated with the serum level of proinflammatory cytokines TNF-α and IL-6 [13]. The present study data revealed that HFD significantly increased IL-6, IL-1β, and TNF-α levels, whereas LPs significantly decreased them. An important signaling pathway in many inflammation-related disorders is the transcription factor NF-κB, which is involved in inflammation, immune response, cell proliferation, differentiation, and survival [38,39,40,41,42]. NF-κB is considered a central link in systemic inflammatory responses and lipid nephrotoxicity caused by lipotoxicity and is mediated by TLR4 to regulate inflammation [43]. HFD activates the TLR4/NF-κB signaling pathway, and the results of this study revealed that LP therapy remarkably down-regulated the level of TLR4 in the TLR4/NF-κB pathway to reduce the expression of NF-κB, thereby reducing the level of inflammatory factors. These findings suggested that LPs inhibit NF-κB mobilization and inflammatory cytokine secretion to alleviate inflammatory stress.

It should be noted that long-term HFD leads to the excessive production of renal ROS, and ROS-induced oxidative stress is another risk factor of renal injury [44]. Hyperlipidemia may down-regulate the antioxidant capacity in tissues and cells, thereby increasing the levels of superoxide free radicals, H_2_O_2_, and MDA and further reducing the activities of T-AOC, SOD, CAT, and GSH-Px in tissues or serum [45,46]. The MDA level in the HFD group was greatly increased and the activities of T-AOC, SOD, CAT, and GSH-Px were significantly decreased. However, oxidative stress caused by HFD was reduced by LPs, and the antioxidant capacity of the kidney was restored. Therefore, LPs may be crucial in restoring the antioxidant capacity of the kidney for treating HFD-induced renal damage. Nrf2, a major regulator of cellular redox homeostasis, is involved in liver fat metabolism and affects blood glucose homeostasis [47,48]. Nrf2 activation can be used to improve HFD-induced hyperglycemia, participate in visceral fat metabolism, and reduce oxidative stress [49,50,51]. The present study results revealed that LP treatment improved Nrf2, HO-1, and NQO1 levels in HFD-induced renal injury, suggesting that LPs protect cells from oxidative stress.

Changes in the type and abundance of intestinal flora may have an impact on how amino acids, lipids, and carbohydrates are metabolized, which may lead to kidney damage. As per the animal model experiments in this study, the association between an inflammatory response driven by physiologic dysregulation and altered lipid metabolism was related to the emergence of obesity-related kidney damage. The healing impact of LPs on HFD was evaluated, and the results revealed that the impact is mediated by controlling fat accumulation, an imbalance in the gut microbiota, and subsequent activation of the gut–kidney axis. It is a comprehensive system that functions in opposition to the initiation of the inflammatory response and includes anti-inflammatory, antioxidant, and other processes.

Mice on high-fat and high-sugar diets exhibited low diversity in gut microbiota, suggesting that these diets have an influence on the colonization and stability of the gut microbiota. The primary mechanisms of renal damage due to endotoxemia and inflammation are bacterial overgrowth and increased intestinal permeability. The HFD and the ND groups had different metagenomic outcomes at the gate level, leading to microbial dysregulation in HFD-fed mice. Microbial dysregulation induced by HFD led to an increase in Gram-negative bacteria and, more importantly, a general decrease in the abundance of all microorganisms [52]. Intestinal microbiome dysregulation was combatted by LPs, which also significantly altered the composition of the gut microbiome at various taxonomic levels and decreased fat accumulation in mice. In HFD-fed mice, LPs improved the *Firmicutes/Bacteroidetes* ratio and enhanced bacterial diversity. According to the research [53], Bacteroides colonizing a host’s stomach may break down a range of plant polysaccharides that are not digested by the host and supply the host with nutrients, which is advantageous to both the host and bacteria. A slight connection between the gut microbiota and central nervous system was also discovered, and inflammation was significantly correlated with the *Bacteroidetes/Firmicutes* ratio [54]. LEfSe analysis confirmed that LPs primarily increased the *Bacteroidales–S24–7 group* and *Lactobacillus* abundance compared with the HFD group. *Bacteroidales–S24–7 group* and *Lactobacillus* are regarded as beneficial organisms in the human body. An increase in species richness can reduce the likelihood of renal inflammation and enhance the body’s tolerance to endotoxins via the strengthening of the intestinal epithelial barrier and prevention of bacterial displacement. LPs can regulate intestinal flora disorder, promote the growth of pertinent beneficial bacteria, and inhibit the formation of pathogenic bacteria.

In the present study, HFD was shown to cause noticeable changes in the composition of intestinal flora. In addition, according to the findings of the functional prediction analysis of the gut microbiota in mice, intestinal disorders had a strong correlation with kidney damage, and the gut microbiota composition changed as a result of long-term HFD. This change can have an impact on kidney function through several pathways such as metabolism, renal cell carcinoma, the renin–angiotensin system, and the transport and catabolism systems, which is in line with Rite’s “gut–kidney axis” theory [23]. The signs of intestinal flora imbalance may also be associated with the occurrence of renal toxicity. Bacterial displacement, intestinal mucosal damage, and changes in metabolites caused by intestinal microbiota dysregulation might offer research insights; however, the precise function of intestinal microbiota dysregulation in kidney injury remains unknown.

## 4. Materials and Methods

### 4.1. Preparation of Muscle Peptides from Monkfish

The preparation method of monkfish fish polypeptides was compared with the previous method [30]. After digestion with E/S neutral protease (2000 U/g) for 5 h at 45 °C, the fat of monkfish fish was removed, and the fish polypeptide LPs (<1 kDa) was obtained via ultra-filtration freeze-drying method with the enzyme removed.

### 4.2. Identification Analysis and Protein Analysis of LPs (<1 kDa)

By following Tang et al.’s method, amino acid content was determined [55]. In brief, a solution of LPs was hydrolyzed at 110 °C with 6 mol/L HCl for 24 h. A sample volume of 1 mL was dried in liquid nitrogen, dissolved in 1 mL buffer solution (pH = 2.2), and analyzed using an amino acid analyzer. To determine the level and proportion of LP peptides, de novo sequencing analysis, the NCBI database, and nanoUPLC-MS/MS were utilized.

### 4.3. Animals and Treatments

C57BL/6J mice were obtained from Hangzhou Ziyuan Experimental Animal Science and Technology Co., Ltd. (Hangzhou, China) with animal production license no. SCXK (ZHE 2019-0004), and Zhejiang Ocean University’s Experimental Animal Ethics Committee (Zhoushan, China) approved the study. Adaptive feeding was performed for a week, and mice were randomly classified into four groups (32 mice in total, 8 per group), including one group that was the ND group and another that followed an HFD. HFD intervention for 8 weeks leads to lipid nephrotoxicity. A previous study revealed a strong dose–effect connection between the antioxidant capacity of LPs and their concentrations and revealed that the cell viability could be effectively restored at a concentration of 200 μg/mL LPs [30]. Therefore, for animal investigations, 100 and 200 mg/kg were employed. HFD mice were classified into three groups HFD (saline), LP-100 (LPs 100 mg/kg per day), and LP-200 (LPs 200 mg/kg per day). Food and water were freely provided to mice in all groups. According to the body mass of mice, LPs were administered daily through gavage feeding. After 4 weeks of therapy, the blood of the mice was collected after ether anesthesia. For testing, the serum was stored at −80 °C after collection. Mouse kidneys were weighed, fixed with 4% paraformaldehyde, and embedded in paraffin.

### 4.4. Detection of Renal Function Index

BUN, CRE, and UA kits (Nanjing Jiancheng Biological Engineering Institute, Nanjing, China) were used to measure the levels of BUN, CRE, and UA, respectively, in the renal tissue, as per the manufacturer’s instructions.

### 4.5. Indices of Oxidative Stress Detection 

The MDA, T-AOC, GSH-Px, SOD, and CAT kits (Nanjing Jiancheng Biological Engineering Institute, Nanjing, China) were used to measure the levels of MDA, T-AOC, SOD, GSH-Px, and CAT, respectively, in the renal tissue, as per the manufacturer’s instructions.

### 4.6. Analysis of Proinflammatory Factors in Renal Tissue

Approximately 10% renal homogenate was obtained using PBS instead of normal saline. TNF-α, IL-6, and IL-1β concentrations in the homogenate were measured using ELISA kits (Elabscience Biotechnology, Inc., Wuhan, China).

### 4.7. Histopathological Analysis

After fixing with 4% paraformaldehyde, the renal tissues were paraffin-embedded. H&E staining, PAS staining, and histological investigation were performed on 4 cm paraffin slices. The slides were imaged using light microscopy at 400× magnification (OlympusCX31, Tokyo, Japan).

### 4.8. Tissue Protein Extraction and Western Blotting

Liquid nitrogen was used to grind kidney tissue, which was then homogenized in RIPA buffer. Western blotting was performed in accordance with the Tang et al. study. [56] Renal protein concentration was measured with a BCA kit (KeyGENbio, Nanjing). The polyacrylamide gel contains 20 µg of protein per well. The following antibodies were used: Keap1 (1:1000, Mouse, Proteintech), Nrf2 (1:2000, Mouse, Proteintech), NQO1 (1:5000, Mouse, Proteintech), HO-1 (1:2000, Mouse, Proteintech), NF-κB (p65) (1:1000, Rabbit, Beyotime), Phospho-NF-κB (p-p65) (1:1000, Rabbit, Beyotime), TLR4 (1:500, Rabbit, Beyotime), and GAPDH (1:100,000, Mouse, Proteintech). Proteins were detected via ECL (ECL, TransGen Biotech, Beijing). Western blotting bands and quantitative analysis were performed using the Fluochem-FC3 system (ProteinSimple, Waltham, MA, USA).

### 4.9. Collection of Mice Feces

Following 16 weeks of feeding (day 1), the fecal samples of mice were collected. The brief procedure was as follows: The mice’s lower abdomen was stimulated to encourage defecation, the anal region was cleaned with medical alcohol, feces were collected in sanitized EP tubes, and the tubes were stored in −80 °C freezer for intestinal flora sequencing.

### 4.10. Intestinal Microflora Analysis 

To analyze the intestinal flora, fecal samples were sent to Shanghai Yuanxin Biomedical Technology Inc. After completing genomic DNA extraction, the primer connector was designed and synthesized. Specific primers (forward 5′-CCTAGGRRBGCASCALGRVGAAT-3′ and reverse 5′-GGACATCNVGGTWTTCTATCCC-3′) were used to amplify the 16s rRNA genes (V3 and V4 hypervariable regions) of different intestinal bacteria. First, PE readings obtained from MiSeq sequencing were split according to the overlapping relationship. Meanwhile, sequence quality was controlled and screened. OTU cluster analysis and species classification analysis were performed after samples were differentiated. The intestinal microbiota of the mice in each group was comprehensively analyzed using α-diversity analysis, β-diversity analysis, and other data analysis methods, and the species abundance information of intestinal flora of mice in each group was obtained. For a deeper understanding of the variations in microbial community richness, Metastats and LEfSe analyses of the intestinal flora of mice were also performed. The association between intestinal flora and kidney damage may also be understood via functional predictive analysis.

### 4.11. Statistical Analysis

The data are reported as mean + standard deviation (SD) using IBM SPSS 19.0 (Ehningen, Germany). The means of each group were compared using one-way ANOVA followed by a Dunnett *t*-test. Statistical significance was defined as *p* < 0.05.

## 5. Conclusions

LPs protected the mice from HFD-induced chronic kidney damage. LPs prevented HFD-induced oxidative stress by inhibiting NF-κB activation, which, in turn, reduced inflammation. Moreover, LPs improved the predominant species of intestinal flora and significantly altered the composition and structure of the intestinal flora in HFD-fed mice. Few effective medications are available to treat HFD-induced CKD. The present study employed LPs extracted from monkfish and demonstrated that LPs may be an effective therapy for HFD-induced kidney injury and offered a fresh perspective on the investigation of HFD-induced kidney injury. The optimization of this polypeptide’s metabolic pathway and dose form have not yet been studied, necessitating further research.

## Figures and Tables

**Table 1:** LP composition (g/100 g) in amino acids.

Amino Acid Species	LPs
Asp	5.82 ± 0.190
Thr	2.83 ± 0.083
Ser	2.89 ± 0.080
Glu	9.75 ± 0.297
Gly	2.53 ± 0.080
Ala	3.45 ± 0.107
Cys	0.00
Val	3.51 ± 0.096
Met	2.42 ± 0.051
lle	2.79 ± 0.079
Leu	5.57 ± 0.152
Tyr	1.81 ± 0.014
Phe	6.19 ± 0.079
Lys	5.48 ± 0.167
His	1.55 ± 0.102
Arg	3.70 ± 0.105
Pro	0.00
HAA	25.74
PCAA	10.73
NCAA	15.59
EAA	28.43

**Table 2:** Effect of LPs on kidney index of mice.

	ND	HFD	LP-100	LP-200
Kidney index (%)	0.011779 #	0.012482 *	0.011972	0.011744 #

* *p* < 0.05 vs. ND group; # *p* < 0.05 vs. HFD group.

**Table 3:** UA, CRE, and BUN levels in mice fed HFD.

	ND	HFD	LP-100	LP-200
BUN (mmol/L)	7.374051 ##	14.4893 **	12.43961 **##	9.910283 **##
CRE (μmol/L)	8.844319 ##	15.07347 **	12.37732 **##	10.31556 ##
UA (μmol/L)	8.701216 ##	18.93794 **	15.35509 **##	9.724888 ##

** *p* < 0.01 vs. ND group; ## *p* < 0.01 vs. HFD group.

**Table 4:** LPs effect on renal MDA, SOD, GSH-Px, T-AOC, and CAT levels in HFD-fed rats.

	ND	HFD	LP-100	LP-200
SOD (U/mgprot)	142.2944 ##	41.63757 **	83.6647 **##	124.3602 *##
CAT (U/mgprot)	7.29797 ##	3.28831 **	5.33356 **##	6.227272 *##
T-AOC (U/mgprot)	64.21501 ##	33.64305 **	47.81102 **##	54.3458 *##
MDA (nmol/mgprot)	3.475465 ##	7.23004 **	5.129147 **##	4.09263 ##
GSH-Px (U/mgprot)	28.39727 ##	9.782904 **	17.85859 **##	26.20776 ##

* *p* < 0.05, ** *p* < 0.01 vs. ND group; ## *p* < 0.01 vs. HFD group.

**Table 5:** Effect of PSCP on the levels of renal IL-1β, IL-6, and TNF-α in the HFD-fed mice.

	ND	HFD	LP-100	LP-200
IL-6 (pg/mL)	89.78946 ##	117.9453 **	102.73 *#	93.06537 ##
IL-1β (pg/mL)	64.47391 ##	99.95587 **	85.46253 **#	72.53305 ##
TNF-α (pg/mL)	26.36301 ##	78.06664 **	64.16955 **#	46.029 **##

* *p* < 0.05, ** *p* < 0.01 vs. ND group; # *p* < 0.05, ## *p* < 0.01 vs. HFD group.

## Data Availability

Not applicable.

## Sample Availability

Samples of the compounds are not available.
